# Current Practices of Medication Plans in Austrian Patients Undergoing Coronary Angiography: An In-Depth Analysis

**DOI:** 10.3390/jcm13113187

**Published:** 2024-05-29

**Authors:** Johannes B. Vogel, Magdalena Neyer, Pascal Elsner, Alexander Vonbank, Thomas Plattner, Christoph H. Saely, Andreas Leiherer, Heinz Drexel

**Affiliations:** 1 Faculty of Medical Sciences, Private University in the Principality of Liechtenstein (UFL), 9495 Triesen, Liechtensteinandreas.leiherer@vivit.at (A.L.); 2 Vorarlberg Institute for Vascular Investigation & Treatment (VIVIT), 6800 Feldkirch, Austria; 3 Department of Internal Medicine I, Academic Teaching Hospital Feldkirch, 6800 Feldkirch, Austria; 4 Medical Central Laboratories, 6800 Feldkirch, Austria; 5 Landeskrankenhaus-Betriebsgesellschaft, Academic Teaching Hospital Feldkirch, 6800 Feldkirch, Austria; 6 Drexel University College of Medicine, Philadelphia, PA 19129, USA

**Keywords:** medication plan, medication list, medication safety, polypharmacy, transition of care, patient safety, medication reconciliation

## Abstract

A complete medication plan (MPlan) increases medication safety and adherence and is crucial in care transitions. Countries that implemented a standardized MPlan reported benefits on patients’ understanding and handling of their medication. Austria lacks such a standardization, with no available data on the issue. **Objective:** This study aimed to investigate the current state of all medication documentations (MDocs) at hospital admission in a population at high risk for polypharmacy in Austria. **Methods:** We enrolled 512 consecutive patients undergoing elective coronary angiography. Their MDocs and medications were recorded at admission. MDocs were categorized, whereby a MPlan was defined as a tabular list including medication name, dose, route, frequency and patient name. **Results:** Out of 485 patients, 55.1% had an MDoc (median number of drugs: 6, range 2–17), of whom 24.7% had unstructured documentation, 18.0% physicians’ letters and 54.3% MPlans. Polypharmacy patients did not have a MDoc in 31.3%. Crucial information as the patients’s name or the originator of the MDoc was missing in 31.1% and 20.4%, respectively. Patients with MDoc provided more comprehensive medication information (*p* = 0.019), although over-the-counter-medication was missing in 94.5% of MDocs. A discrepancy between the MPlan and current medication at admission existed in 64.4%. In total, only 10.7% of our patient cohort presented an MPlan that was in accordance with their current medication. **Conclusion:** The situation in Austria is far from a standardized MPlan generated in daily routine. Numerous MPlans do not represent the current medication and could pose a potential risk for the effectiveness and safety of pharmacotherapy.

## 1. Introduction

Medication errors are among the most frequent adverse events in health care as pharmaceuticals are the most common therapeutic intervention [1,2]. Caused by inappropriate prescribing, dispensing, incompleteness of documentation, transcription errors and patients’ medication-intake behavior this problem is of multifactorial origin. Further, fostered by polypharmacy, multimorbidity, the number of involved healthcare professionals (HP) and increasing transition points of care as a consequence of increasing specialization, medication errors are globally expected to rise further [2].

To counteract this problem, knowledge about patients’ medication is crucial for all stakeholders involved in therapy, including HPs, informal caregivers and patients themselves. Accurate and complete exchange of information on medication is of utmost importance in the medication reconciliation process at transition points of care [3,4] and should encompass what the patient is actually taking—including over-the-counter medication and dietary supplements. This crucial information is often inadequate and HPs have to review multiple medical reports and consult other HPs or relatives to determine this basic information prior to any further treatment.

While the per capita consumption of pharmaceuticals continues to rise across Western nations [5,6], the increasing number of medications prescribed has resulted in a notable surge in hospital admissions due to adverse drug events [7,8,9]. Additionally, social systems are facing a substantial economic burden associated with non-adherence [10,11]. In countries where a standardized medication plan (MPlan) has already been established, several positive effects have been reported. With a MPlan intake errors could be avoided and adhering to prescribed medication became more consistent. Patients demonstrated an enhanced knowledge and understanding of their prescribed medication, including indication and name of the corresponding drug. They could also provide more correct information regarding dosage and dosage frequency e.g., during hospital admission. This led to a heightened sense of subjective self-confidence in handling their medical treatment [12]. 

In Germany, since 2016, patients required to regularly take more than three drugs are entitled to receive a standardized medication plan [13]. In Switzerland efforts are underway to comprehensively implement the “eMediplan” both as a patient-held version (paper and an app for smartphone) and in the patient’s personal electronic health record [14]. A broad consensus was already established with all stakeholders including software manufacturers and initial issuance by certain HP is ongoing [2,15]. In other Central European countries similar developments have been made and some are actively planning to establish a standardized MPlan [16,17,18].

Austria has previously implemented an electronic health record and more recently introduced the electronic prescription system. However, there is currently no information available regarding the development of a standardized MPlan in this means. Consequently, the type and quality of medication documentation (MDoc) used by patients remain unknown. This study aims to provide the first comprehensive analysis of the MDoc and MPlan landscape in Austria, focusing on elective hospital admissions for patients at high risk of polypharmacy.

## 2. Methods

### 2.1. Patients

We investigated a consecutive series of 512 patients between March 2022 and July 2023 at the Academic Teaching Hospital Feldkirch, Austria. All patients were referred for an elective coronary angiography for the evaluation of established or suspected stable coronary artery disease (CAD). They were routinely encouraged to bring a detailed list of all their medications, MDoc (including name, dosage, and frequency), or all their medicines to the appointment in the hospital (brown-bag-review). During the daily clinical admission process the latest medication was documented by a physician at anamnesis following the routine protocol and the brought in MDoc from the patient was copied for further analysis. Physicians assessed the patient’s ability to provide adequate information about their medication during the medical history interview and recorded this using a 4-point Likert scale (Patient can provide comprehensive information—Patient can provide partial information—Patient can only provide inadequate information—Medication cannot be clarified with the patient). Moreover, anthropometric data, questionnaires and possible over-the-counter medication of the patients were investigated during a standardized interview by the study physician. Patients with no medication (*n* = 16) at point of admission were excluded in the further evaluation.

### 2.2. Analysis of Medication Documentation

MDoc was defined as every written documentation (digital, handwritten or printed) patients brought along for admission to the hospital. The in-detail classification of MDoc and their handwritten annotations was conducted by three pharmacists (JBV, MN, PE) based on an adapted Framework Method [19]. The analysed handwritten annotations originated from patients or their informal caregivers themselves and were already present in the MDocs at admission.

The category MPlan was defined as a structured tabular printed list containing the name of the patient. MDocs directed to another physician or MDocs with corresponding labeling were considered physician’s letters. Other handwritten, home-made or printed MDocs without the name of the patient were summarized in the category unstructured documentation. Other documentation comprises digital MDocs on mobile phones, prescriptions or prescription copies and copies of packages. 

The detailed evaluation of the quality of MPlans was conducted based on the criteria of the German standardized MPlan, BMP [20], and the Swiss eMediplan [21]. Detailed information on the items of these standardized MPlans, compared with the investigated items in this analysis, is shown in the Supplementary Materials (Table S1).

### 2.3. Meciation and Discrepancy Analysis

The content of MDocs was reconciled with the medication recorded in the digital anamnesis-protocol upon hospital admission, which was defined as the gold standard. Following Steurbaut et al. any differences between these two documents in drug (name), route, dosage, or frequency were classified as a discrepancy [22]. Furthermore, drug count per patient was calculated and in a more in-depth analysis medication complexity was investigated using the validated German version of the medication regimen complexity index (MRCI) [23,24].

### 2.4. Statistical Analysis

All statistical analyses were performed with the software package SPSS 28.0 for Windows (IBM, Chicago, IL, USA). *p* values below 0.05 were defined as statistically significant. Differences were tested for statistical significance with the Mann-Whitney U test for continuous variables and the Chi-squared test for categorical variables. Figures were generated with Microsoft Office Professional Plus 2021 (Microsoft Corporation, Redmond, WA, USA).

This study followed the STROBE guidelines for reporting cross-sectional studies [25].

## 3. Results

**Figure 1:**
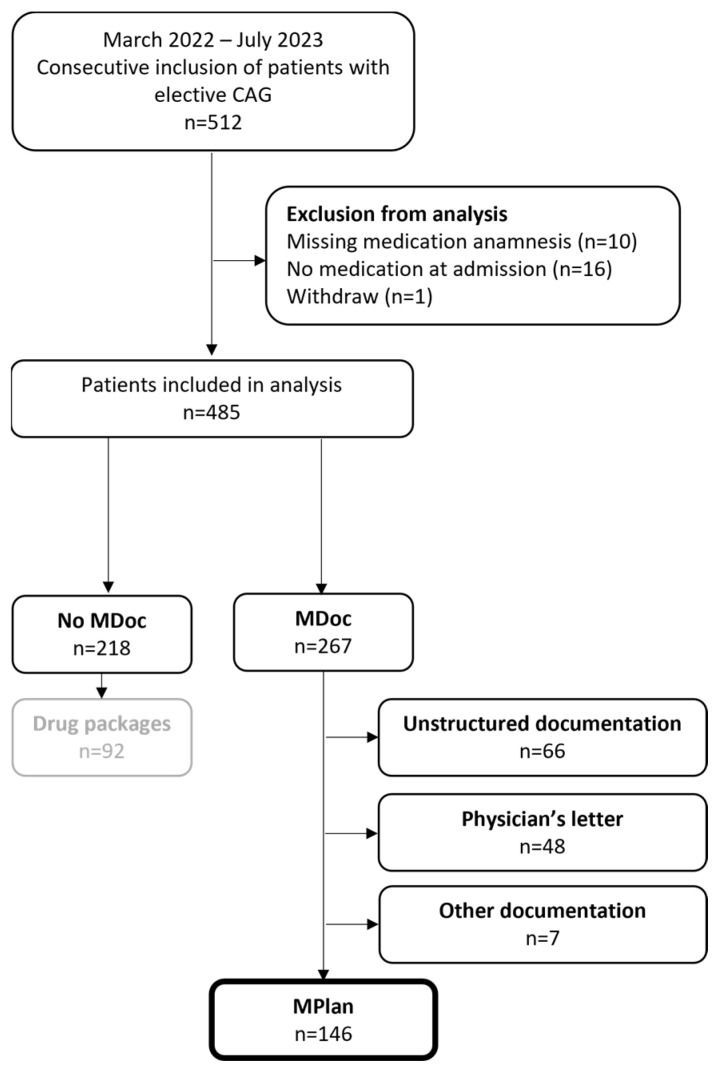
Flow chart of study population. Category “other documentation” comprises documenta-tions on mobile phones (*n* = 1), prescriptions (*n* = 3) and copies of packages (*n* = 3). MDoc—Medication documentation; MPlan—Medication plan.

In total 512 patients were referred for an elective coronary angiography from which 485 patients were included in the analysis. Figure 1 shows the in- and exclusion of patients in detail. Table 1 gives an overview of patients’ baseline characteristics.

### 3.1. Medication Documentation

**Figure 2:**
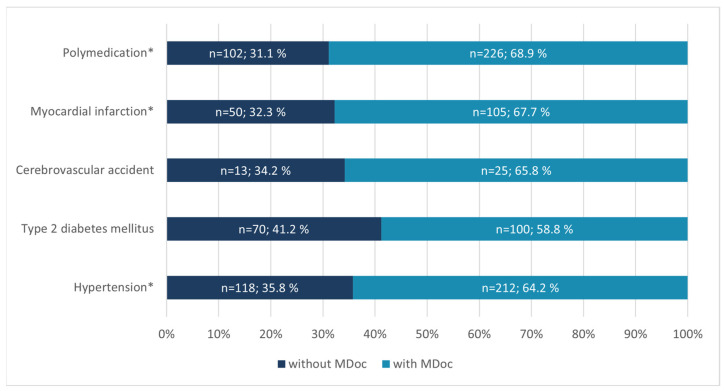
Overview of patient histories and polymedication status in relation to the presence of a MDoc in Austrian CAG-Patients at hospital admission. MDoc—Medication documentation; *—indicates a statistically significant difference (*p* < 0.05) for the marked variable.

At hospital admission 55.3% of patients (*n* = 267) were able to present a MDoc. As shown in Table 1, patients without MDoc were significantly younger in age and had significantly fewer medications per patient compared to the group with MDoc. Among this group, at least 42% (*n* = 92) brought their drug packages to the hospital. A history of severe cardiovascular events or hypertension was associated with a higher likelihood of having a MDoc (*p* < 0.001). Similar results were observed for polypharmacy (*p* < 0.001). Conversely, no statistically significant difference was found for patients with type 2 diabetes mellitus (T2DM) or cerebrovascular accidents. However, approximately one third (31.1% and 32.3%) of all patients on polymedication (≥5 drugs) and all patients with a history of myocardial infarction did not have any form of MDoc available upon admission (Figure 2).

**Figure 3:**
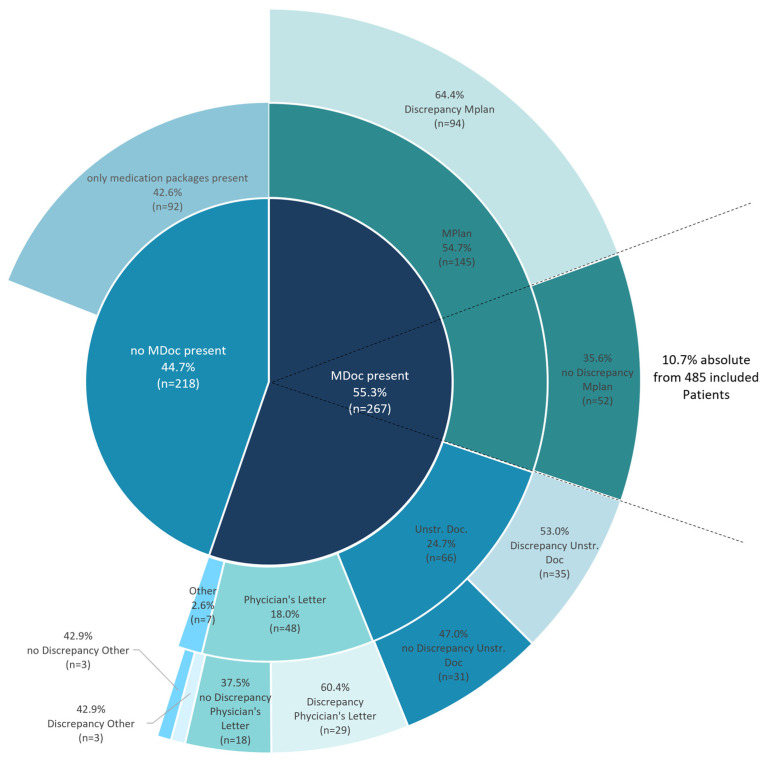
Stacked pie chart overview of MDocs in Austrian CAG-Patients at hospital admission and Further categorization. The category “other” comprises documentations on mobile phones (*n* = 1), prescriptions (*n* = 3) and copies of packages (*n* = 3). MDoc—Medication documentation; MPlan—Medication plan; Unstr. Doc.—Unstructured documentation.

The MDocs patients brought along to the hospital were heterogenous and not standardized. The different categories of MDocs are shown in Figure 3. Whereas more than half of the patients with MDoc (*n* = 146 (54.7%)) had a MPlan, almost 25% of patients (*n* = 66 (24.7%)) used unstructured documentation of their medication. These unstructured MDocs were heterogenous in size and in 65.2% (*n* = 43) written by hand. Some examples of unstructured MDocs are presented in supplementary to highlight the broad variety (Figure S1).

In the assessment by the study physician 9.8% of patients without MDoc vs. 5.7% of patients with MDoc could not give sufficient information to clarify their actual medication during anamnesis (*p* = 0.019).

### 3.2. Medication Plan

In total 145 patients had a MPlan defined as printed, structured, tabular medication report with the name of the patient. Most of the MPlans were issued by specialists (39.3%), followed by hospital physicians (23.4%) and general practitioners (21.4%). In about 80% of MPlans there were no form fields to note information on allergies and contraindication. Further, over-the-counter medication, information on the patients’ general practitioner or the active pharmaceutical ingredient (API) were missing in over 90%, up to nearly 100% (Table 2). No MPlan had a Bar- or QR-Code to use the comprised information digitally.

### 3.3. Discrepancy Analysis

Analyses of inconsistencies between the documents patients brought with them and their current medication revealed discrepancies in 60.3% of MDocs and 64.8% of MPlans. As a result, only 10.7% of patients (*n* = 52) within the study population exhibited accurate MPlans (Figure 3, outermost circle). In a secondary analysis of patients on lipid lowering therapy (LLT) (ATC-Code C10) or on antidiabetic medication (ATC-Code A10) no statistically significant difference in low-density lipoprotein cholesterol (LDL-C) or HbA1C and fasting blood glucose was observed in dependence of discrepancies in their MDoc.

### 3.4. Handwritten Annotations

**Figure 4:**
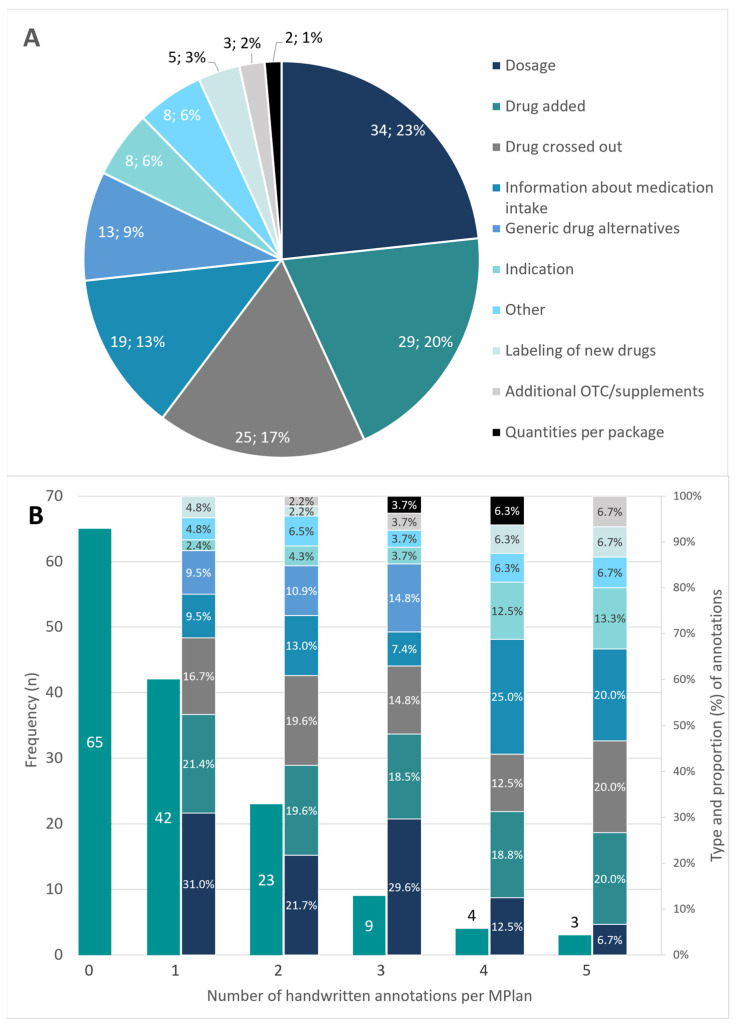
(**A**). Type of handwritten annotations and frequency in total. (**B**). Frequency and number of handwritten annotations per MPlan. Type and proportion of handwritten annotations in context of the number of annotations are shown on the secondary axis; the color code for the annotation types in (**A**) is also valid for (**B**); MPlan—Medication plan; OTC—over-the-counter medication.

More than half of the MPlans (55.5%, *n* = 81) had handwritten adjustments and over 10% (*n* = 16, 11.0%) had three or more different types of corrections. Figure 4 states frequency of handwritten annotations and groups the annotations by type. The distribution of the different types of correction in correspondence with the total number of handwritten annotations per MPlan is shown on the secondary axis in Figure 4B.

### 3.5. Self-Reported Co-Medication

When asked for OTC-medication 45.6% of patients (*n* = 221) reported to take additional co-medication (mean = 1.3, *n_min_* = 1, *n_max_* = 4). Patients stated not only OTC-substances (*n* = 82), nutritional supplements (*n* = 58) or homeopathic substances (*n* = 8) but also drugs that are only available on prescription (*n* = 73). This co-medication was represented in 5.5% of the MPlans.

## 4. Discussion

Our research uncovers crucial aspects of type and quality of patient held MDocs in general and specifically MPlans among Austrian patients: (i) the supply situation with MDocs is significantly dependent on the patients’ medical condition but is generally inadequate; (ii) only one out of ten patients is able to present a MPlan that is in accordance with the medication actually being taken at an elective hospital admission; (iii) quality of MDoc is extremely heterogenous, offering numerous potential sources for medication errors and reaching from paper scraps to professional administered MPlans; (iv) essential information on a MDoc such as the name of the patient and the date of issue are often missing and lacking actuality of a vast majority of MDocs appears often confirmed by numerous handwritten annotations; (v) documentation of OTC medications is almost non-existent in current MDocs, while prescription-only drugs repeatedly are stated as “OTC” by patients.

### 4.1. General Supply Situation

Despite the detailed analysis of MDocs and MPlans shown in the results section, our data indicates that 26.0% (*n* = 126) of patients presenting for an elective hospital admission have absolutely no source of information on their medication other than their personal disclosure in variable quality. Anyhow, another 19.0% at least presents their medication packages for a brown-bag review at admission. This makes medication anamnesis difficult and time-consuming, impacts medication safety, impairs adherence and puts risk on the effectiveness of pharmacotherapy. About one third of polypharmacy patients, a highly vulnerable group for adverse drug reactions, cannot present any written information about their pharmacotherapy. In our study 55% of patients have a MDoc and more than half of all MDocs are already in the format of a structured MPlan on which the patient and the very basic characteristics of their pharmacotherapy are evident. Despite that we have observed a significant difference in the availability of MDocs depending on the ailment. Patients with cardiovascular disease are better supplied with a MDoc opposed to patients with type 2 diabetes mellitus (T2DM). Given that both conditions are chronic, involve multiple medications and require regular medical check-ups, more care should be taken in supplying T2DM patients with written information on their medication regimen.

### 4.2. Actuality and Completeness

Based on the analysis of the handwritten annotations, it is to be assumed that more than half of the patients (55.5%) are not satisfied with their MPlan. Either it comprises too little information, or it lacks actuality and is completed by the patients or the informal caregivers themselves. This finding of our study compares favorably with those reported from a study conducted in New York, where the authors found at least one annotation in over half of patient’s medication lists [3]. Although clinical significance of the annotations was not rated in our investigation, they seem to be important for the patients. The fact that the three annotation types dosage, drug added and drug crossed out are accountable for over 60% of all manual corrections indicates that actuality of most of the plans might be lacking. Further 20% of the annotations regard information on medication intake and generic drug alternatives, whereby especially the proportion of the intake-annotations strongly rises in MPlans with more than three annotations. All these obviously missing data would systematically be covered by a standardized medication plan, as the BMP or the eMediplan.

Unexpectedly, unstructured MDocs exhibit the fewest discrepancies in comparison to data from medication anamnesis. One possible explanation could be that patients who prepare documentation themselves do this more accurately than physicians with limited time per patient [26]. Waltering et al. similarly reported less discrepancies in documentation written by patients and relatives in contrast of plans issued by physicians [27]. Hence, it is to hypothesize that patients should be given the chance to get involved in managing their pharmacotherapy. Physician’s letters and MPlans were surprisingly found to be among the least reliable sources of information, with discrepancy rates of 60.4% and 64.4%, respectively. These rates are barely acceptable for clinical practice. This finding is slightly above the results from other studies (60.4% [22], 53.6% [28]) 54.4% [29]) but confirms validity also for Austria. On several MPlans the date of issue was missing, therefore it was impossible to evaluate if the MPlans were outdated or not. The risk of discrepancies in MPlans rises with time passed from the date of issue and was reported in literature to be 2.6-fold higher for plans older than one month [30]. According to our secondary analysis presence or absence of discrepancies between the MDoc and the medication anamnesis at hospital admission has no impact on LDL cholesterol or HbA1C and fasting glucose for patients on LLT or antidiabetic medication, resepctively. This surprising finding could be explained with the fact, that the discrepancies in our study were ascertained for the medication regimen in whole, not for LLT or antidiabetic medication in specific. Hence, this secondary finding is not to be overestimated, and further studies on this issue are needed.

In patients’ self-reported co-medication not only OTC-medication or nutritional supplements are stated, but also a high number of diverse drugs only obtainable with prescription. However, these drugs are mostly not represented in the MPlan, especially when not administered orally [31]. Knowledge of co-medication could offer important additional information on comorbidities (e.g., laxatives for indigestion) or prevent possible interactions with novel medication (e.g., multivalent cations from mineral supplements). Moreover, some OTC-drugs can influence laboratory data of patients (e.g., biotin clinically significant distorts troponin) or could be a hint on possible prescription cascades [30]. Physicians and pharmacists should be aware of the fact that additional medication is not documented in 94.5% of the MPlans in Austria today.

### 4.3. Implications on Daily Clinical Practice

In the light of the contemporary challenges posed by the aging population within the healthcare system, there seems to be increasingly limited time available per patient. This constraint has been identified as a driving factor to polypharmacy. In Austria the allocated time is a mere five minutes, placing it among the lowest of the investigated countries. Irving et al. reported that “An average of five minutes may be the limit below which consultations amount to little more than triage and the issue of prescriptions” [26]. In this context, sharing of medication information could significantly save administratively wasted time at transition points in healthcare systems, consequently enhancing quality and can be facilitated by the use of patient-held medication records [1]. Our study further confirms that patients with a written MPlan can provide significantly more information on their medication [13] and therefore could possibly contribute to an efficient anamnesis. But in fact, our investigation reveals that about one third (31.1%) of all patients on polymedication (≥5 drugs) do not have any kind of documentation about their medicines. This poses significant challenges for clinical practice when implementing additional pharmacological interventions, particularly considering that the incidence of drug-drug interactions reaches approximately 40% among patients taking five medications and exceeds 80% when using 7 or more drugs. Ensuring continuous medication safety for this vulnerable group appears extremely challenging through this means [32].

In a survey by Dormann et al. over one third of physicians and pharmacists stated they could identify adverse drug reactions, contraindications and potential drug interactions based on a standardized MPlan in over one out of five patients [33] whereas nearly 80% found a higher guideline conformance when treatment regimens were documented within a structured MPlan [34]. Even though there are proven benefits of standardized MPlans, which not only enhance patients’ medication handling and perception but also particularly facilitate work at transition points of care—the groundbreaking advantage is offered by the digital usability of the structured information within the plan. In our study no MPlan had any barcode or QR-Code to use the comprised information digitally. The great benefit of this would be the risk minimalization of transcribing errors and potentially decreasing time for medication anamnesis at transition points of care [4]. Transferring of data could be automated and every involved healthcare professional would be able to receive the complete medication and medication-relevant information of the patient within seconds. Background interaction checks and with the help of Clinical-Decision-Support-Software monitoring permanently for the safest medication (e.g., dosage at renal insufficiency, multiple-prescription or allergy-drug-checks) would be easily possible. To ensure data-readability trough all medical software systems a nationwide standard is needed.

### 4.4. Strengths and Limitations

This study has several strengths. First, our sample size was above many other investigations on this topic [3,22,28,30]. Another notable strength of this study is that every type of MDoc that was brought by the patient was analysed. Therefore, our results represent data from the real life clinical daily business. As patients had no commonalities except referral for an elective CAG, a broad cross-section of the population, including patients with different co-morbidities, was investigated. This makes our data highly transferrable to the general situation regarding medication documentation. Another important strength is that our study was not limited to the evaluation of the MDoc itself, but also investigated the discrepancies between the documentation and the actual medication as a quality parameter. Further, this is the first ever analysis of medication plans in Austria and can therefore play a crucial role in a pre-post study after future implementation of a standardized MPlan. One potential limitation of our study is that it was conducted at a single center and did not include electronic MDocs, however numbers of electronic MDocs were so low that this limitation appears negligible. The strengths and weaknesses of the study data, as well as the opportunities and threats resulting from this study, are further explored through a SWOT analysis [35] presented in Table 3.

### 4.5. Implications for Policy

Policymakers face the challenge of ensuring a sustainable healthcare system for an aging population. This involves improving efficiency, automating processes and above all—optimizing resource utilization. To address the growing issue of polypharmacy and ensure medication safety, a national strategy for a standardized medication plan, a central pillar in any eHealth solution, is urgently needed to safely transfer medication information to all stakeholders including patients themselves.

## 5. Conclusions

This investigation revealed that the status quo in Austria is far from a standardized MPlan, generated in daily routine and up-to-date for patients. Numerous MPlans do not represent the current medication and essential information is often missing in clinical practice. Thirty percent of polypharmacy patients cannot show any kind of MDoc, while patients with a history of chronic conditions, particularly T2DM, are generally inadequately provided with MDoc. This could pose a potential risk for the effectiveness and safety of pharmacotherapy. A standardized medication plan can be seen as a fundamental element for ensuring a safe and beneficial medication process and is integral to any eHealth solution.

## Figures and Tables

**Table 1:** Baseline characteristics of patients (*n* = 485) with and without medication documentation (MDoc) at hospital admission.

	Patients		
	without MDoc (*n* = 218)	with MDoc (*n* = 267)	*p*-Value
Age (years)	65.4 ± 11.5	70.2 ± 10.1	<0.001
Male gender	165 (75.7%)	191 (72.1%)	0.369
Native German speaking	194 (93.3%)	239 (93.4%)	0.969
Currently smoking	42 (19.3%)	37 (14.0%)	0.121
Body mass index (kg/m^2^)	23.5 ± 4.2	23.7 ± 4.6	0.759
Systolic BP (mm Hg)	136.0 ± 40.2	135.5 ± 49.5	0.325
Diastolic BP (mm Hg)	79.4 ± 23.4	77.4 ± 26.2	0.728
**Medication**			
Drugs per patient	4.8 ± 2.9	7.0 ± 2.8	<0.001
Over-the-counter medication per patient	0.5 ± 0.7	0.7 ± 0.8	0.062
MRCI	11.5 ± 7.4	17.5 ± 8.3	<0.001
Drug-count ≥ 3	173 (79.4%)	263 (98.5%)	<0.001
Polymedication (≥5 drugs)	102 (46.8%)	226 (84.7%)	<0.001
**History of**			
Myocardial infarction	50 (22.9%)	105 (38.5%)	<0.001
Cerebrovascular accident	13 (6.0%)	25 (9.5%)	0.148
T2DM	70 (32.1%)	100 (37.7%)	0.198
Hypertension	118 (54.4%)	212 (80.3%)	<0.001
**Laboratory Values**			
LDL-C (mg/dL)	91.9 ± 41.2	75.3 ± 34.2	<0.001
HDL-C (mg/dL)	53.2 ± 17.3	51.25 ± 14.0	0.420
Fasting Glucose (mg/dL)	116.5 ± 35.8	122.3 ± 38.5	0.084
HbA1c (%)	6.0 ± 0.7	6.2 ± 0.7	0.002

Characteristics of patients were assessed at baseline. For categorical variables absolute numbers (*n*) and relative proportions (%) are presented. For continuous variables mean ± standard deviation is presented. *p*-Values were produced from Mann-Whitney-U-Test or Chi-squared Test. T2DM—type 2 diabetes mellitus (according to the definition of the American Diabetes Association); BP—blood pressure; MRCI—Medication Regimen Complexity Index; LDL-C—low-density lipoprotein-cholesterol; HDL-C—high-density lipoprotein-cholesterol; HbA1c—hemoglobin A1c.

**Table 2:** Summary of available items in the different categories of MDoc. Investigated items were selected according to established standardized MPlans in Germany [20] and Switzerland [21].

Items	MPlan (*n* = 146)	Unstructured Doc. (*n* = 66)	Physician’s Letter (*n* = 48)	Other (*n* = 7 *)	MDoc in Total (*n* = 266 *)
Date of issue	122 (84.1)	16 (24.2)	14 (29.2)	3 (50)	156 (58.9)
Name (incl. first and last name)	146 (100)	16 (24.2)	46 (95.8)	3 (50)	211 (79.6)
Date of birth	125 (85.6)	10 (15.2)	30 (83.0)	3 (50)	177 (66.8)
Insurance number	36 (24.7)	7 (10.6)	3 (6.3)	3 (50)	49 (18.5)
Allergies/contraindications	28 (19.4)	1 (1.5)	4 (8.3)	0	33 (12.6)
Drug name	146 (100)	65 (98.5)	47 (97.9)	6 (100)	264 (99.6)
Active pharmaceutical ingredient	1 (0.7)	2 (3.0)	1 (2.1)	0	4 (1.5)
Quantity and quantity unit	142 (97.3)	47 (71.2)	47 (97.9)	3 (50)	239 (90.2)
Posology (simplified dosage scheme with times)	144 (98.6)	48 (72.7)	47 (97.9)	6 (85.7)	245 (91.8)
Indication	15 (10.3)	8 (12.1)	0	0	23 (8.7)
Further information	61 (52.1)	8 (14.5)	28 (58.3)	0	97 (45.1)
Self-medication (e.g., OTC)	11 (7.5)	2 (3.0)	0	1 (16.7)	14 (5.3)
Machine-readable Data Code	0	1 (1.5)	0	3 (50)	4 (1.5)
Originator of Documentation					
Not documented	21 (14.4)	58 (87.9)	2 (4.2)	4 (66.7)	86 (32.5)
General practitioner	34 (12.8)	2 (3.0)	0	0	34 (12.8)
Specialist	71 (26.8)	1 (1.5)	11 (22.9)	2 (33.3)	71 (26.8)
Hospital	68 (25.7)	1 (1.5)	33 (68.8)	0	68 (25.7)
Other health facility	6 (2.3)	3 (4.5)	1 (2.1)	0	6 (2.3)
Patients’ general practitioner	7 (4.8)	2 (3.0)	0	0	9 (3.4)

Given as n (%); * digital plan on phone not further analysed. MPlan—Medication Plan; Doc.—Documentation; OTC—over-the-counter.

**Table 3:** SWOT analysis of the study data; SWOT—Strengths, Weaknesses, Opportunities, Threats; MDoc—Medication documentation; MPlan—Medication plan; CAG—coronary angiography; OTC—over-the-counter medication.

	Strengths	Weaknesses
Internal	**Comprehensive data collection:** The dataset includes detailed information on patients, their medical history, medication and laboratory parameters, MDoc and MPlan, discrepancies between MDoc and medication anamesis at hospital admission, handwritten annotations, co-medication, OTCs and interviews, providing a thorough basis for analysis. **Real-world data:** Being based on actual clinical practice, the dataset reflects real-world scenarios, enhancing the applicability of the findings. **National data:** Significant differences among healthcare systems across nations (e.g., competencies, digitalization levels, professional responsibilities) hinder the transferability of study data. Thus, national data plays an important role in addressing specific healthcare challenges. **Volume of data:** The large dataset and the long period of time offer more robust insights into this topic. **Standardization and interoperability:** The data is standardized to the best possible extent according to established research methods and classifications, as well as according to standardized MPlans from other countries. This facilitates comparison across different studies and might enhance compatibility with other healthcare research databases (e.g., for meta-analyses) **Multidisciplinary team:** In order to have complete information and to cover the most crucial aspects from different point of views on this issue, the study was conducted by a multidisciplinary healthcare professional team consisting of community pharmacists, hospital and IT-pharmacists, general physicians, cardiologists, endocrinologists and medical laboratory scientists.	**Biases:** The dataset might contain biases, such as over-representation of certain patient groups due to the study’s monocentric nature. Referral for CAG should not, but can differ from geographical regions in Austria. **Temporal relevance:** Data may become outdated once eHealth solutions are established nationwide. Then, new data will be necessary to assess the situation. **Documentation variability:** Huge differences in how medications are documented (MDoc) had to be overcome through classification and standardization. Although this has been done with great diligence through multiple researches and according to scientific standards, it might be possible that there is a variability between different research groups which affects data comparability.
	Opportunities	Threats
External	**Improved patient outcomes:** This data can help leading to better patient care strategies. **Enhanced clinical guidelines:** “High-risk spots” through the whole medication documentation process could be identified through this study. This could help to enhance hospital internal processes (e.g., systematically addressing OTC medication and supplements in a standardized medication history anamnesis; MPlan at discharge for all patients taking 3 or more drugs). **Policy development:** Findings can support healthcare policy changes aimed at improving patient safety and health system efficiency at a macro-economic level. **Economic efficiency:** Adequate sharing of medication information can reduce healthcare costs by decreasing medication errors, hospital readmissions, adverse drug events and by accelerating anamnesis processes at every transition point of care. **Integration with technology:** The dataset supports promotion of implementing eHealth solutions. **Research collaboration:** The dataset can foster collaborations among researchers, potentially broaden the project’s research impact and leading to innovations in patient safety.	**Data misinterpretation:** Misinterpretation of the study data is a risk that can lead to incorrect conclusions and actions. **Regulatory changes:** Changes in healthcare regulations or data protection might impact how data can be used and shared in future. **Economic fluctuations:** macro-economic factors, such as healthcar funding cuts or economic downturns, could hinder the utilization of this data for further developing and implementing improvements in (digital) healthcare.

## Data Availability

The data that support the findings of this study are available from the corresponding author upon reasonable request.

## Supplementary Materials

The following supporting information can be downloaded at: https://www.mdpi.com/article/10.3390/jcm13113187/s1, Figure S1: Anonymized examples (*n* = 7) of unstructured medication Documentation (MDoc); Table S1: Comparison of items of standardized medication plan (MPlan) in Switzerland [21] and Germany [20] with investigated items in non-standardized MPlan in Austria.
