# Supplementary material for: Current Practices of Medication Plans in Austrian Patients Undergoing Coronary Angiography: An In-Depth Analysis
Source: J Clin Med. 2024 May 29;13(11):3187. doi: 10.3390/jcm13113187 (PMC11172892; doi:10.3390/jcm13113187)
Supplement: Supplementary file 1 [file jcm-13-03187-s001.zip › jcm-2993197-supplementary.pdf]

Article – Supplementary

Current Practices of Medication Plans in Austrian Patients Undergoing Coronary Angiography: An In-Depth Analysis

Johannes B. Vogel [1,2], Magdalena Neyer [1,2], Pascal Elsner [1,2], Alexander Vonbank [1,2,3], Thomas Plattner [1,2,3], Christoph H. Saely [1,2,3], Andreas Leiherer [1,2,4] and Heinz Drexel [1,2,4,5,6,*]

[1] Faculty of Medical Sciences, Private University in the Principality of Liechtenstein (UFL), 9495 Triesen, Liechtenstein; andreas.leiherer@vivit.at (A.L.)
[2] Vorarlberg Institute for Vascular Investigation & Treatment (VIVIT), 6800 Feldkirch, Austria
[3] Department of Internal Medicine I, Academic Teaching Hospital Feldkirch, 6800 Feldkirch, Austria
[4] Medical Central Laboratories, 6800 Feldkirch, Austria
[5] Landeskrankenhaus-Betriebsgesellschaft, Academic Teaching Hospital Feldkirch, 6800 Feldkirch, Austria
[6] Drexel University College of Medicine, Philadelphia, PA 19129, USA
* Correspondence: heinz.drexel@vivit.at

SUPPLEMENTARY

1. Supplementary Tables

Table S1: Comparison of items of standardized medication plan (MPlan) in Switzerland (21) and Germany (20) with investigated items in non-standardized MPlan in Austria.

Items of the eMediplan in Switzerland*	Items of standardized MPlan (BMP) in Germany*	Analysed items in investigated MPlan in Austria
	**Header**	
Title (Medication Plan)	Title (Medication Plan)	
Page	Page	
Total numbers of pages	Total numbers of pages	
Date of issue		Date of issue
	**Patient**	
First name	First name	Name (incl. first and last name)
Last name	Last name	
Date of birth	Date of birth	Date of birth
Gender	Gender	
Address		
Patient's language		
Contact Data (Phone, Email)		
Patient ID (Insurance Number)		Insurance Number
	**Medical Data**	
Risk categories (organ insufficiencies, Allergies, Diabetes…)	Allergies<br>Creatinin	Allergies, Contraindications
Pregnancy (First day of last menstruation, Time of gestation)	Pregnancy	
Anthropometrics (Weight Height)	Anthropometrics (Weight Height)	
	**Medication & Posology**	
Tradename of the drug	Tradename of the drug	Name of the drug (+Dosage Form)
Dosage Form	Dosage form	
Active pharmaceutical ingredient	Active pharmaceutical ingredient	Active pharmaceutical ingredient
Quantity and quanitity unit	Quantity and quantity unit	Quantity and quantity unit
Taking reason	Taking reason	Indication
Application instructions	Additional information (Method of Administration)	Further Information
Self-medication	Self-medication	Self-medication (OTC, NEM)
Method of Administration	Method of Administration	
Substitution allowed		
From date		
To date		
Reserve medication	Drugs at specific times (e.g. reserve-medication)	
Posology (Daily, FreeText, Single, Cyclic, Sequence, Timed Dosage, complex taking times, pause) incl. simplified version of taking times	Posology (simplified dosage scheme and taking times)	Posology (simplified dosage scheme with times)
Prescripted by (e.g. physician, pharmacist etc.) (for each drug separately)		
QR-Code (containing the complete information of the eMediplan for further digital use)	2D-Barcode (containing the complete information of the BMP for further digital use)	Machine-readable Data-code
	**Healthcare Professional**	

GLN (Registered number)		
First name	First name	Originator of MPlan
Last name	Last name	
Address (Postcode, City)	Address (Postcode, City)	
	Contact details (Phone, Mail)	
	Date and Time of issue	Date of issue
		General practitioner

* Items were adapted for comparison by the authors.
MPlan – Medication Plan

2. Supplementary Figures

Figure S1: Anonymized examples (n=7) of unstructured medication Documentation (MDoc).

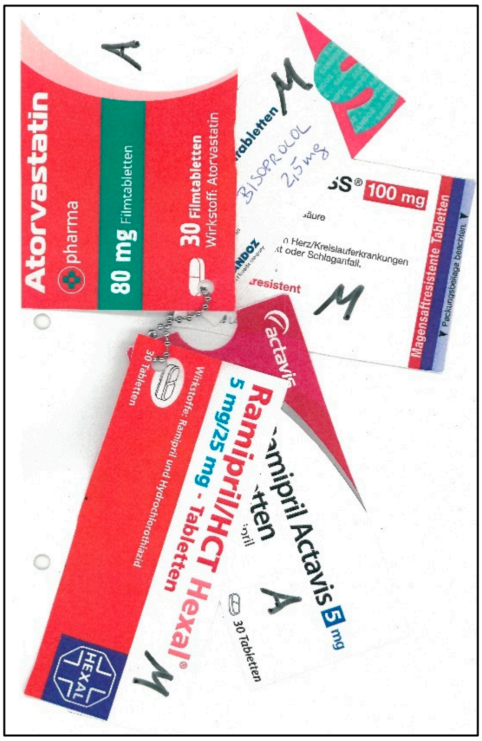

4A: a creative pocket version of bundled medication packages. The letters "A" and "M" reference intake times, although "M" in German is not unequivocal, as it can stand for both "Morgen" (morning) and "Mittag" (noon), hence posology remains unclear.

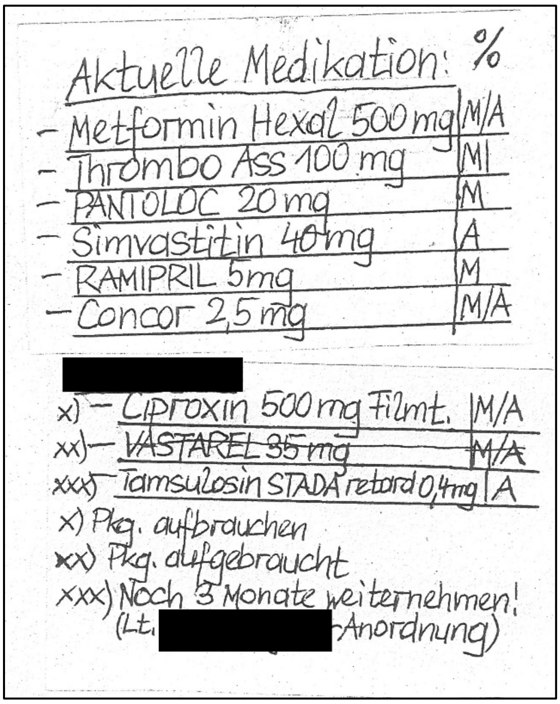

4B: illustrates a self-crafted medication schedule in credit card format. Similarly, the intake times are not explicit. This MDoc includes additional rudimentary information such as 'use up package' or 'take for another 3 months,' even in the absence of specific dates.

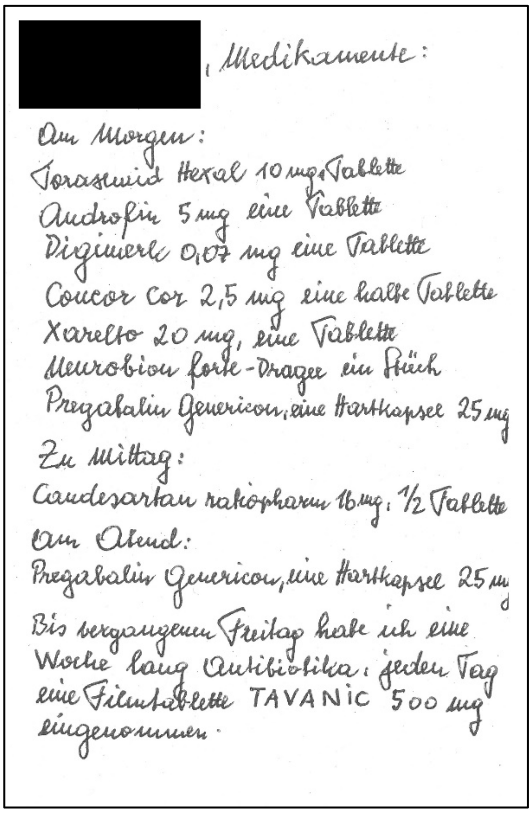

[redacted], Medikamente:

Am Morgen:
Torasemid Hexal 10 mg, Tablette
Androfin 5 mg eine Tablette
Digimerk 0,07 mg eine Tablette
Concor Cor 2,5 mg eine halbe Tablette
Xarelto 20 mg, eine Tablette
Neurobion forte-Dragee ein Stück
Pregabalin Genericon, eine Hartkapsel 25 mg
Zu Mittag:
Candesartan ratiopharm 16 mg: 1/2 Tablette
Am Abend:
Pregabalin Genericon, eine Hartkapsel 25 mg
Bis vergangenen Freitag habe ich eine Woche lang Antibiotika, jeden Tag eine Filmtablette TAVANIC 500 mg eingenommen.

4C: handwritten MDoc in cursive script, the listed times of day are arranged vertically, complicating overall readability and information comprehension.

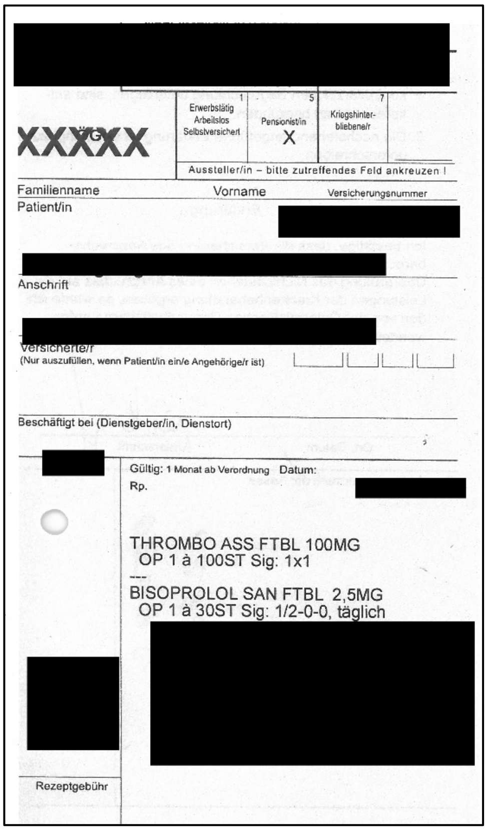

[redacted]

1 Erwerbstätig Arbeitslos Selbstversichert | 5 Pensionist/in X | 7 Kriegshinterbliebene/r

XXXXX

Aussteller/in – bitte zutreffendes Feld ankreuzen !

Familienname Vorname Versicherungsnummer
Patient/in [redacted]

[redacted]

Anschrift

[redacted]

Versicherte/r
(Nur auszufüllen, wenn Patient/in ein/e Angehörige/r ist)

Beschäftigt bei (Dienstgeber/in, Dienstort)

[redacted]

Gültig: 1 Monat ab Verordnung Datum:
Rp. [redacted]

THROMBO ASS FTBL 100MG
OP 1 à 100ST Sig: 1x1

BISOPROLOL SAN FTBL 2,5MG
OP 1 à 30ST Sig: 1/2-0-0, täglich

[redacted]

Rezeptgebühr

4D: prescription copy.

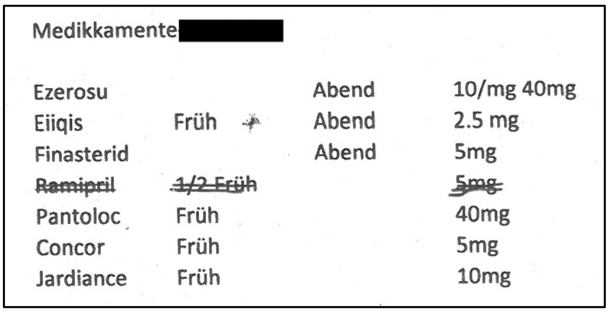

Medikkamente [redacted]

Ezerosu		Abend	10/mg 40mg
Eiiqis	Früh	Abend	2.5 mg
Finasterid		Abend	5mg
~~Ramipril~~	~~1/2 Früh~~		~~5mg~~
Pantoloc	Früh		40mg
Concor	Früh		5mg
Jardiance	Früh		10mg

4E: computer generated MDoc with handwritten corrections.

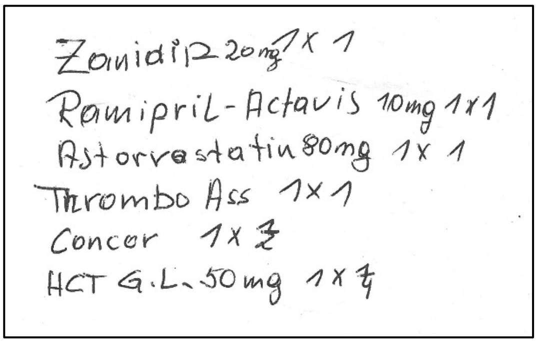

Zanidip 20mg 1 x 1
Ramipril-Actavis 10mg 1 x 1
Astorvastatin 80mg 1 x 1
Thrombo Ass 1 x 1
Concor 1 x [illegible]
HCT G.L. 50 mg 1 x [illegible]

4F: handwritten MDoc, the intake times are entirely unclear, as well as the prescribed dosage for some medications.

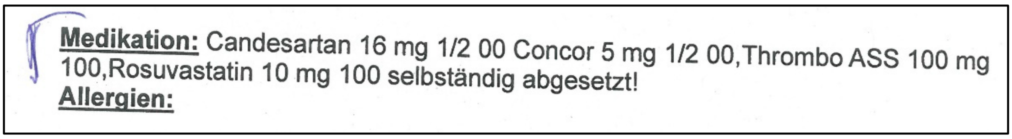

Medikation: Candesartan 16 mg 1/2 00 Concor 5 mg 1/2 00,Thrombo ASS 100 mg 100,Rosuvastatin 10 mg 100 selbständig abgesetzt!
Allergien:

4G: excerpt from a physician's letter, notable is the way the posology is written, which inconsistently separates simplified intake times and could be prone to transcription errors.
